# Packed School Lunch Food Consumption: A Childhood Plate Waste Nutrient Analysis

**DOI:** 10.3390/nu15051116

**Published:** 2023-02-23

**Authors:** Jack R. Thomas, Derek Hanson, Ashley Chinnan-Pothen, Christine Freaney, Jill Silverman

**Affiliations:** Department of Nutrition Science and Wellness, Farmingdale State College, 2350 Broadhollow Rd, Farmingdale, NY 11735, USA

**Keywords:** packed school lunch, NSLP, plate waste, nutrient intake, elementary school, New York, sugar sweetened beverage, sodium, fiber, cholesterol

## Abstract

Packed school lunch consumption remains a sparsely studied aspect of childhood nutrition. Most American research focuses on in-school meals provided through the National School Lunch Program (NSLP). The wide variety of available in-home packed lunches are usually nutritionally inferior compared to the highly regulated in-school meals. The purpose of this study was to examine the consumption of home-packed lunches in a sample of elementary-grade children. Through weighing packed school lunches in a 3rd grade class, mean caloric intake was recorded at 67.3% (32.7% plate waste) of solid foods, while sugar-sweetened beverage intake reported a 94.6% intake. This study reported no significant consumption change in the macronutrient ratio. Intake showed significantly reduced levels of calories, sodium, cholesterol, and fiber from the home-packed lunches (*p* < 0.05). The packed school lunch consumption rates for this class were similar to those reported for the regulated in-school (hot) lunches. Calories, sodium, and cholesterol intake are within childhood meal recommendations. What is encouraging is that the children were not “filling up” on more processed foods at the expense of nutrient dense foods. Of concern is that these meals still fall short on several parameters, especially low fruit/vegetable intake and high simple sugar consumption. Overall, intake moved in a healthier direction compared to the meals packed from home.

## 1. Introduction

Health and nutrition studies on packed school lunches typically split their focus on either preschool, elementary, or high school intake, each requiring its own research focus. The purpose of this study was to examine the consumption of home-packed lunches in a group of 3rd grade elementary children. Few studies have empirically examined home-packed school lunches for nutritional content. However, a multitude of researchers have examined the caloric/nutritional content of hot meals provided in schools [1,2,3]. The National School Lunch Program (NSLP) dominates most research on school lunches. NSLP focuses on delivering nutritious food at reduced or no cost to families in need [4]. Over 90% of schools accept this federal program, which typically provides an estimated 30 million meals a day in the United States [5]. That leaves tens of millions of meals provided to children by their families every school day. Estimates place the number of home-packed lunches at U.S. schools at around 40% of all school meals, with their packed content found to have a higher caloric value and a lesser nutritional value than in-school lunches [6]. All NSLP-participating schools are obligated to make all their lunch offerings meet minimum nutrition/caloric standards and be healthy in nature [7]. Because schools meet these strict NSLP nutrition parameters, parent-packed lunches have been called inferior on many levels [8,9,10].

The selection and preparation of these home meals are complex issues. With no guiding regulations on what to pack, home meals provide all the food and calories a child consumes during a typical lunch period, which should result in 25 to 30 percent of their daily food intake [11]. Multiple food choices exist for packed school lunch preparation but are absent from the in-school meals because that food is provided by the school on a cycle menu. Prominent variables influencing the packing of a child’s lunch include parent/child communication [12], geographic region, economic status of the school/region/family, ethnic makeup of the school, and school administrative policies [13].

Because parent-packed lunches are tailored towards a student’s likes, consumption is thought to be greater than in school lunches [14]. One area of concern with home-packed meals is their high energy density, especially sugar-sweetened beverages, chips, and baked goods [15]. Childhood obesity research occasionally discusses using both in-school and packed school lunches to help understand/control body weight. Historically, lunchtime dietary intake has become secondary to physical activity [16]. A 2018 review found 18 weight-focused studies used physical activity interventions, and only 3 contained dietary means to control childhood obesity [17].

### 1.1. Weighing Lunch Plate Waste (New Sub Section)

It is a challenge to assess dietary intake among children since information for school food intake is often derived from self-reported questionnaires, collected by memory, dietary recall, or from food photograph/visual analysis [18]; none of these are direct measurements. The recall error in some dietary intake methods has been estimated at more than 30 percent [19]. Therefore, most articles on intake rely on large subject numbers to normalize the data and stress the training of data collection personnel to help reduce this large potential error. Visual interpretation of school plate waste, though validated, was found inferior to the more laborious scale weighing of leftovers [20]. Plate waste measurement is the direct weighing of food. It is considered the most accurate method of nutrient intake because no memory or portion estimates are used [21]. While there are advantages to memory recalls, questionnaires, and the visual interpretation of school lunches, weighing provides the most specific information by overcoming estimations that depend on student memory or observation [22].

### 1.2. Parenting

The primary responsibility for home-packed lunch content is with the parent/adult. The amount of time and effort put into meal preparation is often limited, resulting in processed and prepackaged food items being the easy choice. This is especially true for working families that have both parents employed and those with multiple children. The primary role of in-school NSLP lunches is to meet minimum nutritional standards. The main reason for packing lunches, according to parents, is not nutritional but to meet the child’s taste preferences [23].

Parents may well be concerned about nutrition, but they do not have to abide by federal or school guidelines. Most often, the main concerns are for their children to maintain a healthy body weight and to eat what they pack [24]. Estimates place the number of home-packed lunches at U.S. schools at around 40% of all school meals, and they have determined their total content, as packed, to have a higher caloric value and lesser nutritional value than in-school lunches [25,26]. However, what is packed and what is eaten are two very different things. Because of their wide variety, home meals present extra challenges to research. The lack of rigorous examination of consumption leaves the actual nutritional intake of packed lunches in doubt [27].

Preparation of school lunches must fit into the family schedule and work with family dynamics both inside and outside the home. Some parent food preparation behavior is best described as unconcerned when it comes to constructing school lunches. This minimal effort is associated with higher calorie, sodium, and fat content [28]. Other parents show coercive control practices by pressuring their children to eat healthy at both home and school. Some parents address their meal preparation efforts as simply providing access to healthy foods at home and in school lunches [29].

In 2017, preschooler home-packed lunches were reported not to provide consistently adequate nutrients [30]. After nursery school and the first couple of years of elementary school, parents become accustomed to the general eating likes and dislikes of their children [31], and they buy food accordingly. Regardless of the age of the child, lunch leftovers/waste returning home is viewed as a negative reinforcing factor for parents buying food and packing meals [32]. It influences future lunch preparation. Finding food returned from school sends the message not to buy more of that food in the future. It is noteworthy that one study on children and eating habits said that up to 30% of children throw away food to avoid conflict with their parents [33].

### 1.3. School Policies and Plate Waste

Intrusive school policy and poor food quality have been shown to be the two biggest contributors to plate waste for in-school lunches [34]. These same policies also influence plate waste for home-packed lunches. Poor communication among administrators, food service staff, health educators, and teachers reduces healthy eating behavior for all school children [35]. A longer scheduled lunchtime at school increases the amount of food consumed [36]. The later in the afternoon schools serve lunch, the greater the consumption of both in-school and packed lunches because the kids are hungrier [37,38]. Rules such as having lunch before recess (exercise), not having enough time to eat, not sharing unopen foods, and not talking among peers all lead to increased food waste [38,39,40,41].

## 2. Materials and Methods

This research followed a group of 3rd grade children from a suburban Long Island, New York, parochial school for 5 consecutive days (*n* = 118). Recruitment was complete and consistent for all student-packed lunches brought from home. Lunches were collected as the children entered the classroom over a one-week period. These meals were taken, labeled with individual identification stickers, and weighed in grams for each individual food item (pre-weight). All packed lunches were then returned to the classroom before lunch. During lunch, children were observed from a distance to ensure no sharing of foods occurred. Children were informed to throw nothing out after eating lunch but to take everything left over to the nearby research table. A lunchroom table was established, with trash cans placed behind the researchers. All home-packing third graders handed the entire lunch bag, including all wrapping and food waste, to the researchers. Using the identification numbers, the leftover items were reweighed (post-weight), and the lunch containers were returned to the classroom. This direct weighing avoided misreporting eaten food. Weights outside of three standard deviations were considered outliers; no outliers were detected in the sample weights.

Water and calorie-free liquids were weighed but categorized individually from caloric liquids for separate statistical analysis (non-caloric). Any child who purchased prepackaged food from the school cafeteria, such as prepackaged chips, snacks, candy, or cookies, had that food included in the study by weighing individual leftovers against an unused representative package weight. Children with both a packed lunch and supplemented in-school food entrees were not used in this study with the goal of avoiding in-school lunch influence. A Nutritionist Pro (Axxya systems) dietary analysis module software was used to convert individual food pre/post weights into nutrient intake information.

### Data Analysis

Percentages of consumption were calculated from the total food items pre-weighed minus the weight of each leftover food item, including all wrapping material. Sandwiches and other combination items were reported as single-entry items for analysis. All weights were obtained directly by the authors of this study. Descriptive statistics of frequency, mean, standard deviation, and confidence intervals were calculated for all variables. A one-tailed paired *t*-test was performed to compare consumption within home-packed lunches. Pre/post mean differences were considered to be statistically significant at *p* ≤ 0.05. The mean decrease in food weight was determined for energy, macronutrients, water, sugar-sweetened beverages, sodium, fiber, and cholesterol.

## 3. Results

A total of 118 meals were weighed. Not all students were present or consistently packed a daily lunch, resulting in a mean of 23.6 meals/day analyzed. Baseline characteristics of the sample were male students that represented 39.1%, with 60.9% being female. Ages ranged from 8 to 9 years old. Measurements were calculated for the percentage of foods eaten and various nutritional content consumed. The average packed meal arrived with 639.6 kcal; when consumed, the meal averaged 209.1 kcal less, a 32.7% decrease, at 430.6 kcal (*p* = 0.00001) (Table 1). Using a 1550 kcal daily intake as a reference, the children averaged 27.8% of their daily energy requirement with lunch [42].

### 3.1. Fluid Intake and Sugar Sweetened Beverages

A fluid intake analysis showed bottled water was packed with 44% of the lunches, and 92% of that water was consumed with lunch. Sugar-sweetened beverages (usually fruit-based juices) were present in 61% of the lunches (94.6% consumption) (Table 1).

### 3.2. Macronutrient Intake

The macronutrients of carbohydrates, lipids, and proteins all showed no significant percentage difference between what was packed and what was eaten. Carbohydrates changed from 58.1% to 59.5%, lipids from 28.9% to 27.0%, and proteins from 13.3% to 13.4% (Table 2). Packed versus consumed lunches showed an overall 1.4% increase in carbohydrates, a 1.9% decrease in lipids, and a 0.1% increase in proteins.

### 3.3. Sodium Intake

Sodium in packed lunches recorded a mean of 1112.3 ± 1480.3 mg per lunch and was significantly reduced through packed lunch consumption to 635.8 ± 284.2 mg (−57.2%) (*p* < 0.05). Using the Institute of Medicine’s current guideline of 1200 mg sodium intake (aged 4–8) from lunches, students met 53.0% of their USDA daily recommendation from lunch intake (Table 3).

### 3.4. Fiber Intake

The fiber in packed lunches averaged 3.32 ± 1.79 g. Actual fiber consumption was 2.16 ± 0.89 g, with a 65.1% consumption rate (*p* < 0.05). Based on an averaged fiber recommendation for male and female children aged 6–11 of 12.85 g of fiber/day [43], these children ate an average of 16.8% of their daily fiber recommendation at lunch.

### 3.5. Cholesterol Intake

Cholesterol in packed lunches averaged 31.7 ± 27.4 mg cholesterol. The actual consumed cholesterol in lunches was 23.7 ± 22.9 mg. This 83.4% consumption rate was a significant decrease in cholesterol (*p* < 0.05). Conway et al. reported a similar middle school cholesterol intake average of 32.6 mg of cholesterol and a significant difference with seventh graders eating more than 6th graders [44].

### 3.6. Sugar Sweetened Beverage Intake

Daily recommendations call for no more than 10% of total calories from sugar-sweetened beverages, such as fruit drinks [45]. Based on the previous 1550 kcal per day energy recommendation, the lunches’ weighed intake represented a mean of 13.2% of daily total simple sugar calories across the entire sample of students (Table 4). A better representation of intake from sugary fruit-based juice liquids was to exclude the students that only consumed water from this calculation. The 66% of sugary fruit-juice beverage drinkers increased their calorie intake from simple sugar drinks to 19.7% of their total lunch calories.

## 4. Discussion

### 4.1. Plate Waste Weighing and Calories

Approximately 186,000 children in New York attend private schools outside of New York City [46]. This research examined home-packed lunches at one of those private schools in a third-grade elementary class on Long Island. It did not estimate intake; it weighed plate waste to better understand childhood dietary intake and to make comparisons to national childhood dietary references on select parameters. There are important nutritional disparities in intake recorded between estimated and weighed childhood lunches. One study revealed that when estimating food intake, the children reported eating 17% of non-observed food items but only reported 67% of the observed items [47]. All forms of estimated dietary assessment have been criticized with respect to accuracy [48,49]. The direct weighing of the food ensures accurate reporting of all consumed foods and serves as an important methodological consideration for future research on school lunch food intake. Through weighing, an average of 67.3% of the packed lunch food was eaten by the children. This rate of consumption is comparable to that found for in-school hot lunches, at approximately 70% [50,51,52]. Regardless of whether packed or purchased, school lunch intake has implications for total daily energy intake and childhood obesity [53]. Using age-specific USDA calorie recommendation ranges, an appropriate caloric intake of 1150 kcal was selected for a reference daily energy intake [54]. Packed meals as they arrived at school met 41.3% of the daily energy intake and would be considered high in calories. The actual consumption of these lunches recorded a lower mean of 27.8% daily energy intake. Packed lunches, as eaten, presented a moderate range of calories for a typical child’s lunch.

### 4.2. Macronutrient Distribution

Although the caloric reduction of packed meals was evident, nutrient distribution remained constant. Parent-packed meals arrived at school with 58% carbohydrate, 29% lipid, and 13% protein. Lunches as eaten by the students demonstrated a similar distribution of 60%, 27%, and 13% (carbohydrate, lipid, and protein). These results represent a typical energy distribution of nutrients for a healthy child’s diet. A meal analysis on preschool daycare children by Romo-Palafox et al. found similar child macronutrient intakes in this study at 56, 31, and 15% carbohydrates, lipids, and proteins, respectively [30].

This packed lunch macronutrient distribution falls within basic childhood nutrition recommendations [55]. Importantly, no significant macronutrient composition difference was detected, indicating the children were eating a portion of each food item packed instead of consuming all of select foods and nothing of others. A scenario that did not happen was that the children entirely ate the higher-calorie processed foods and left large amounts of the nutrient-dense foods as waste. This study showed the food waste was evenly distributed among all packed food items, excluding liquids, which were consistently consumed in the 90th plus percentile.

Previous behavioral research on parental food choices in preschool lunch packing supports this recorded “grazing” consumption of food items [56]. It has its basis in the parent/child communication of a home-packed meal [57]. The parental role in preparing a packed lunch for an elementary school child cannot be underestimated. Parent/child communication will always be an important factor in the contents of a child’s packed lunch [58,59]. As food waste returns home each school day, that negative feedback continually informs the parents of the child’s food likes and dislikes. In-school or “hot” lunch children have limited food choices and throw away their food leftovers before reaching home. In packed school lunches, independent of nutrition content, if the child has shown a past tendency to eat specific foods, that behavior will continue in the future [59]. If the child likes everything in the lunch, they avoid nothing. It also explains the observation of very few fruits and vegetables in the packed lunches. Other than prepackaged, single serving containers of apple sauce and mixed-type fruit cups, no fresh fruit other than an occasional banana was recorded. Reasons for low fruit and vegetable intake are beyond the scope of this study but would likely include parent/child communication of likes and dislikes as well as parents seeing uneaten foods returned home, resulting in a negative reinforcement to exclude these types of foods.

### 4.3. Fluid Intake and Sugar Sweetened Beverages

The liquid portion of the packed school lunches revealed relatively higher consumption rates than for solid foods. Bottled water was present in 44% of the packed lunches. The same children brought water consistently throughout the study. Packed lunch bottled water consumption averaged 92%. Bottled water size varied from 8 to 16 ounces, and regardless of size, it was usually completely consumed by the children. For the in-school lunches, the federal government requires access to water at lunchtime for all students and not to restrict the sale of milk to children [60]. No children with packed lunches took water or milk provided by the school in jugs/cups/cartons on the front tables. Because water lacks calories, its weight was excluded from calorie and macronutrient calculations. The presence of bottled water in a packed school lunch was considered a healthy eating choice.

Sugar-sweetened beverages have been a negative factor in school lunches and related to weight gain in children [61]. The added simple sugar intake recommendation is set by the Dietary Guidelines for Americans at less than 10% of total calories [54]. Although simple sugars were not analyzed for all individual food items, liquid simple sugar content in the form of sugary sweetened beverages was recorded as part of the packed lunch pre- and post-weighing. These sugar-sweetened beverages were recorded in 61% of the packed lunches, with a 94.6% consumption rate (Table 2). Normally, carbonated sweetened sodas are included as a simple sugar source in the analysis of a packed school lunch. However, a school policy prevented families from packing carbonated sodas for lunch, and none were recorded for this home-packed lunch sample group. Overall, average sugar-sweetened beverages contributed 13.2% of total packed lunch calories and slightly exceeded the recommendation of less than 10%. For a more applicable analysis, water drinkers were removed from the analysis. For the results of only those children consuming sugar-sweetened beverages, 19.7% of calories came from those liquids (Table 3). The sugar-sweetened beverage lunches were found to contain almost double the amount of recommended simple sugar upon the removal of the water-containing lunches. This simple sugar intake is a minimum representation, as only liquids were analyzed for sugar content. The solid foods were not individualized for simple sugar calories and data should be interpreted only as sugar-sweetened beverages and sugar calories. This 94.6% consumption rate for 61% of the lunches relates this sample’s high sugar-sweetened beverage intake to the risk variable of childhood obesity [62].

### 4.4. Sodium Intake

This study examined children ages 8 to 9. The current transitional regulation standard for in-school lunch sodium intake, as issued by the National School Lunch Program, for the 2023 to 2024 school year is 1225 mg for 6- to 8-year-olds [63]. The recorded average sodium intake from packed school lunches for this group of children was below the “final rule” target amount of sodium intake. The 635.8 ± 284.2 mg average sodium intake of these children meets the strictest federal standards and would be described as a healthy lunchtime intake of sodium.

The sodium targets require in-school lunch programs to meet ever stricter sodium limitations and give schools several years to meet those standards. The most restrictive sodium school lunch reduction mandate for lunches is less than 640 mg [64]. The recorded average sodium intake from the packed school lunch for this group of children was below the amount of sodium intake imposed on the NSLP in-school lunches. Cohen, Richardson, Roberto, and Rim suggest that a lower sodium intake, as seen in their research on elementary and middle school lunches, may have a negative relationship with simple sugar intake [65]. Although complete simple sugar content was not calculated for this study, the sugar-sweetened beverage calories were excessive and would support such a relationship.

A State of Washington parent-packed school lunch study in 2014 used photography with plate waste weight validation and reported school lunch sodium content was 931.8 mg, with consumed sodium at 746.4 mg [66]. This study’s packed lunch sodium intake of 635.8 mg was similar. One caveat of the Washington State study was that these schools were all participating in the Healthier U.S. School Challenge, which provided and encouraged healthy food choices for all the children. This presents a potential confounding variable for their packed lunch intake.

### 4.5. Fiber Intake

Most research on fiber intake focuses on adults and not children. National intake data show most U.S. children do not meet fiber recommendations [67]. This lack of child-specific fiber research is demonstrated by the existence of several different fiber recommendations for children. The FDA’s label guide for childhood fiber is 12 g/1000 kcal consumed. Current research shows children and adolescents only consume approximately half of the suggested 25 g of fiber per day [68]. Consistent with earlier school lunch studies, the fiber intake for this study was low at 2.16 ± 0.89 g. These findings provided 8.6% toward meeting that daily fiber recommendation. Although there was a significant numerical reduction in fiber intake of −1.6 g (*p* < 0.05), the amount of food containing this amount of fiber was small. The starting fiber content of these meals met 13.3% of the fiber recommendations. This is not unexpected because some of the best fiber sources, such as fruits and vegetables, were very low in the packed lunches. A Canadian study on packed lunch content for 7–10 year-old students reported 9.5 g fiber intake through visual food intake observation [69].

### 4.6. Cholesterol Intake

Cholesterol intake from the packed lunches is in agreement with several previous childhood lunch studies, as reported at 23.7 mg of cholesterol [70,71]. The 2015–2020 Dietary Guidelines for Americans removed the cholesterol recommendation of 300 mg/day and replaced it with the guidance of eating as little as possible. For comparison, the old 300 mg/day level was used in percentage calculations. Home-packed lunches were within this recommendation [72]. The “as packed” lunch value for cholesterol was 31.7 mg, and the eaten value was 23.7 mg of cholesterol, with a 74.8% consumption rate, meeting 10.6% of the old daily cholesterol recommendation.

## 5. Summary

This research on packed school lunches, conducted on Long Island in New York, counters several common misrepresentations that home-packed school lunch construction and consumption are of a consistently lesser nutritional value than their in-school alternatives. Regardless of in-school or packed meals, the direct intake of food needs to be considered. Through direct plate waste measurement, the children who ate packed school lunches fell well within calorie recommendations and presented a healthy macronutrient distribution of carbohydrate, lipid, and protein consumption. The macronutrient pre-post consistency showed the children evenly consumed each item packed and did not eat one or two select foods, leaving others untouched. These lunches were reported as being within range for sodium and cholesterol but high in simple sugar and low in fiber. Plate waste weight measurement remains an integral component for determining actual dietary intake. More research on the American and global nutritional quality of home-packed school foods at various ages is important for gaining a better understanding of how to improve childhood nutrition.

## 6. Limitations

Data from this study were obtained from a small sample of 3rd grade children enrolled in a northeastern (New York) suburban parochial school on Long Island and should not be generalized beyond that specific environment to different demographic, socioeconomic, or ethnic population compositions in the United States or globally. The itemized weighing of the after-lunch plate waste required immediate measurement. Any weight recording error or missed plate waste would alter the dataset. The food collection logistics at the end of the lunch period (signal) prevented researchers from individually observing each student’s complete handling of the waste collection. Some paper or food plate waste may have inadvertently avoided researcher collection through outside trash receptacles.

## Figures and Tables

**Table 1:** Packed lunch content vs. actual energy and liquid intake (*n* = 118).

	Kilocalories M ± SD	CI 95%	Water	Sugar-Sweetened Beverage
Packed lunch	639.6 ± 142	613.98–665.22	Found in 44% of lunches	Found in 61% of lunches
Actual intake	430.6 ± 88.2	414.69–446.51	92% consumed	94.6% consumed
Consumption difference	−209.1 kilocalories −32.7% decrease *			

* Indicates a significant paired *t*-test difference: t (118) = 14.34, *p* < 0.00001.

**Table 2:** Packed lunch macronutrient content vs. actual consumption (*n* = 118).

	Carbohydrate % M ± SD	CI 95%	Lipids % M ± SD	CI 95%	Protein % M ± SD	CI 95%
Packed lunch	58.1 ± 14.6%	55.46–60.73	28.9 ± 11.0%	26.92–30.88	13.3 ± 7.8%	11.89–14.71
Actual intake	59.5 ± 14.7%	56.45–61.75	27.0 ± 11.5%	24.92–29.07	13.4 ± 7.6%	12.03–14.77
Consumption Difference	1.4% increase		1.9% decrease		0.1% increase	

**Table 3:** Packed school lunch content versus actual intake of select health-related nutrients (*n* = 118).

	Sodium M ± SD	CI 95%	Fiber M ± SD	CI 95%	Cholesterol M ± SD	CI 95%
Packed lunch	1112.3 ± 1480.3 mg	845.21–1379.45	3.32 ± 1.79 g	3.00–3.64	31.7 ± 27.4 mg	26.76–36.64
Actual intake	635.77 ± 284.2 mg	584.49–687.05	2.16 ± 0.89 g	1.99–2.32	23.7 ± 22.9 mg	19.57–27.83
Consumption difference	−476.5 mg t (118) 3.34 *p* < 0.05		−1.16 g t (118) 2.267 *p* < 0.05		−8 mg t (118) 2.38 *p* < 0.05	

**Table 4:** Packed school lunches meeting nutrient recommendations (*n* = 118).

Childhood USDA Recommended Intakes	Sodium 1800 mg (Aged 9–13)	Sugar-Sweetened Beverages. Limit 10% Total Calories (Including Water Drinkers) (*n* = 118)	Sugar-Sweetened Beverages. Limit 10% Total Calories (Excluding Water Drinkers) (*n* = 66)
Actual intake	35.3% of recommended	13.2% of total calories	19.7% of total calories

## Data Availability

Some restrictions apply to the availability of these data. Even though the data are not individualized, they do deal with children as a sample group. Our institution reserves the right to accept or refuse requests. Data are available from the primary author, Jack Thomas, at thomasj@farmingdale.edu with university approval.

## References

[1] Rees G.A., Richards C.J., Gregory J. (2008). Food and nutrient intakes of primary school children: A comparison of school meals and packed lunches. J. Hum. Nutr. Diet..

[2] Farris A.R., Misyak S., Duffey K.J., Davis G.C., Hosig K., Atzaba-Poria N., McFerren M.M., Serrano E.L. (2014). Nutritional Comparison of Packed and School Lunches in Pre-Kindergarten and Kindergarten Children Following the Implementation of the 2012–2013 National School Lunch Program Standards. J. Nutr. Educ. Behav..

[3] Joyce J.M., Rosenkranz R.R., Rosenkranz S.K. (2020). Evaluation of Variability in Dietary Quality of School Lunches Meeting National School Lunch Program Guidelines by Socioeconomic Status and Rurality. Int. J. Environ. Res. Public Health.

[4] Golino G., Ralston K., Guthrie J. (2021). Participation Trends for Full Price Meals in the National School Lunch Program. Appl. Econ. Perspect. Policy.

[5] United States Department of Agriculture NSLP Fact Sheet (2023). United States Department of Agriculture. www.ers.usda.gov/topics/food-nutrition-assistance/child-nitrition-programs/national-school-lunch-program/.

[6] Johnston C.A., Moreno J.P., El-Mubasher A., Woehler D. (2012). School Lunches and Lunches Brought from Home: A Comparative Analysis. Child. Obes..

[7] Andreyeva T., Tripp A.S., Schwartz M.B. (2015). Dietary Quality of Americans by Supplemental Nutrition Assistance Program Participation Status. Am. J. Prev. Med..

[8] Hur I., Burgess-Champoux T., Reicks M. (2011). Higher Quality Intake From School Lunch Meals Compared with Bagged Lunches. ICAN Infant Child Adolesc. Nutr..

[9] Crepinsek M.K., Gordon A.R., McKinney P.M., Condon E.M., Wilson A. (2009). Meals Offered and Served in US Public Schools: Do They Meet Nutrient Standards?. J. Am. Diet. Assoc..

[10] Peckham J.G., Kropp J.D., Mroz T.A., Haley-Zitlin V., Granberg E.M. (2019). Selection and consumption of lunches by National School Lunch Program participants. Appetite.

[11] Gola A.A.H., Figueroa H., Bardin S., Gearan E. (2021). Reaching a Vulnerable Population: Why Adolescents Participate in the National School Lunch Program. J. Adolesc. Health.

[12] Lindquist K., Mann G., Farris A., Gordon K., Misyak S. (2021). Parent Perspectives of Packing School Lunches. J. Hunger Environ. Nutr..

[13] Bardin S., Washburn L., Gearan E. (2020). Disparities in the Healthfulness of School Food Environments and the Nutritional Quality of School Lunches. Nutrients.

[14] Hubbard K.L., Must A., Eliasziw M., Folta S.C., Goldberg J. (2014). What’s in Children’s Backpacks: Foods Brought from Home. J. Acad. Nutr. Diet..

[15] Taylor J.C., Sutter C., Ontai L.L., Nishina A., Zidenberg-Cherr S. (2019). Comparisons of school and home-packed lunches for fruit and vegetable dietary behaviours among school-aged youths. Public Health Nutr..

[16] Kim Y., Son K., Kim J., Lee M., Park K.H., Lim H. (2023). Associations between School Lunch and Obesity in Korean Children and Adolescents Based on the Korea National Health and Nutrition Examination Survey 2017–2019 Data: A Cross-Sectional Study. Nutrients.

[17] Martin A., Booth J.N., Laird Y., Sproule J., Reilly J.J., Saunders D.H. (2018). Physical Activity, Diet and Other Behavioural Interventions for Improving Cognition and School Achievement in Children and Adolescents with Obesity or Overweight. Cochrane Database Syst. Rev..

[18] Baxter S.D., Guinn C.H., Smith A.F., Hitchcock D.B., Royer J.A., Puryear M.P., Collins K.L., Smith A.L. (2016). Children’s school-breakfast reports and school-lunch reports (in 24-h dietary recalls): Conventional and reporting-error-sensitive measures show inconsistent accuracy results for retention interval and breakfast location. Br. J. Nutr..

[19] Warren J.M., Henry C.J.K., Livingstone M.B.E., Lightowler H.J., Bradshaw S.M., Perwaiz S. (2003). How well do children aged 5–7 years recall food eaten at school lunch?. Public Health Nutr..

[20] Liz Martins M., Cunha L.M., Rodrigues S.S.P., Rocha A. (2014). Determination of plate waste in primary school lunches by weighing and visual estimation methods: A validation study. Waste Manag..

[21] Kubena K.S. (2000). Accuracy in dietary assessment: On the road to good science. J. Am. Diet. Assoc..

[22] Lindke A.R., Smith T.A., Cotwright C.J., Morris D., Cox G.O. (2022). Plate Waste Evaluation of Plant-Based Protein Entrees in National School Lunch Program. J. Nutr. Educ. Behav..

[23] Vollmer R.L. (2019). Parental feeding style changes the relationships between children’s food preferences and food parenting practices: The case for comprehensive food parenting interventions by pediatric healthcare professionals: VOLLMER. J. Spec. Pediatr. Nurs..

[24] Hawthorne D.L., Neilson L.J., Macaskill L.A., Luk J.M.H., Horner E.J., Parks C.A., Salvadori M.I., Seabrook J.A., Dworatzek P.D. (2018). Parental Reports of Lunch-Packing Behaviours Lack Accuracy: Reported Barriers and Facilitators to Packing School Lunches. Can. J. Diet. Pract. Res..

[25] Tugault-Lafleur C.N., Black J.L., Barr S.I. (2017). A Systematic Review of Methods to Assess Children’s Diets in the School Context. Adv. Nutr..

[26] Sweitzer S.J., Briley M.E., Roberts-Gray C., Hoelscher D.M., Staskel D.M., Almansour F.D. (2011). How to Help Parents Pack Better Preschool Sack Lunches: Advice from Parents for Educators. J. Nutr. Educ. Behav..

[27] Minaya S., Rainville A.J. (2016). How Nutritious are Children’s Packed School Lunches? A Comparison of Lunches Brought from Home and School Lunches. J. Child Nutr. Manag..

[28] Chen B., Kattelmann K., Comstock C., McCormack L., Wey H., Bowne M., Meendering J. (2022). Identifying Food Parenting Practices From Comprehensive Home Environment Survey. J. Nutr. Educ. Behav..

[29] Roberts-Gray C., Briley M.E., Ranjit N., Byrd-Williams C.E., Sweitzer S.J., Sharma S.V., Palafox M.R., Hoelscher D.M. (2016). Efficacy of the Lunch is in the Bag intervention to increase parents’ packing of healthy bag lunches for young children: A cluster-randomized trial in early care and education centers. Int. J. Behav. Nutr. Phys. Act..

[30] Romo-Palafox M.J., Ranjit N., Sweitzer S.J., Roberts-Gray C., Byrd-Williams C.E., Briley M.E., Hoelscher D.M. (2017). Adequacy of Parent-Packed Lunches and Preschooler’s Consumption Compared to Dietary Reference Intake Recommendations. J. Am. Coll. Nutr..

[31] Sabinsky M.S., Toft U., Sommer H.M., Tetens I. (2019). Effect of implementing school meals compared with packed lunches on quality of dietary intake among children aged 7–13 years. J. Nutr. Sci..

[32] Johnson C.M., Bednar C., Kwon J., Gustof A. (2009). Comparison of Nutrient Content and Cost of Home-Packed Lunches to Reimbursable School Lunch Nutrient Standards and Prices. J. Child Nutr. Manag..

[33] Zhao C., Panizza C., Fox K., Boushey C.J., Byker Shanks C., Ahmed S., Chen S., Serrano E.L., Zee J., Fialkowski M.K. (2019). Plate Waste in School Lunch: Barriers, Motivators, and Perspectives of SNAP-Eligible Early Adolescents in the US. J. Nutr. Educ. Behav..

[34] Chriqui J.F., Pickel M., Story M. (2014). Influence of School Competitive Food and Beverage Policies on Obesity, Consumption, and Availability: A Systematic Review. JAMA Pediatr..

[35] Cho H., Nadow M.Z. (2004). Understanding Barriers to Implementing Quality Lunch and Nutrition Education. J. Community Health.

[36] Burg X., Metcalfe J.J., Ellison B., Prescott M.P. (2021). Effects of Longer Seated Lunch Time on Food Consumption and Waste in Elementary and Middle School–age Children: A Randomized Clinical Trial. JAMA Netw. Open.

[37] McLoughlin G.M., Edwards C.G., Jones A., Chojnacki M.R., Baumgartner N.W., Walk A.D., Woods A.M., Graber K.C., Khan N.A. (2019). School Lunch Timing and Children’s Physical Activity During Recess: An Exploratory Study. J. Nutr. Educ. Behav..

[38] Hunsberger M., McGinnis P., Smith J., Beamer B.A., O’Malley J. (2014). Elementary school children’s recess schedule and dietary intake at lunch: A community-based participatory research partnership pilot study. BMC Public Health.

[39] Getlinger M.J., Laughlin C.V.T., Bell E., Akre C., Arjmandi B.H. (1996). Food Waste is Reduced when Elementary-School Children Have Recess before Lunch. J. Am. Diet. Assoc..

[40] Grao-Cruces A., Velázquez-Romero M.J., Rodríguez-Rodríguez F. (2020). Levels of Physical Activity during School Hours in Children and Adolescents: A Systematic Review. Int. J. Environ. Res. Public Health.

[41] Price J., Just D.R. (2015). Lunch, recess and nutrition: Responding to time incentives in the cafeteria. Prev. Med..

[42] Griffen A.S. (2016). Height and calories in early childhood. Econ. Hum. Biol..

[43] Kranz S., Brauchla M., Slavin J.L., Miller K.B. (2012). What Do We Know about Dietary Fiber Intake in Children and Health? The Effects of Fiber Intake on Constipation, Obesity, and Diabetes in Children. Adv. Nutr..

[44] Conway T.L., Sallis J.F., Pelletier R.L., Powers H.S., Marshall S.J., Zive M.M., Elder J.P. (2002). What Do Middle School Children Bring in Their Bag Lunches?. Prev. Med..

[45] Nguyen M., Jarvis S.E., Tinajero M.G., Yu J., Chiavaroli L., Mejia S.B., Khan T.A., Tobias D.K., Willett W.C., Hu F.B. (2023). JSugar-sweetened beverage consumption and weight gain in children and adults: A systematic review and meta-analysis of prospective cohort studies and randomized controlled trials. Am. J. Clin. Nutr..

[46] Domanico R. (2020). A Statistical Profile of New York’s K-12 Educational Sector: Race, Income, and Religion. https://files.eric.ed.gov/fulltext/ED604326.pdf.

[47] Baxter S.D., Thompson W.O., Litaker M.S., Guinn C.H., Frye F.H.A., Baglio M.L., Shaffer N.M. (2003). Accuracy of Fourth-Graders’ Dietary Recalls of School Breakfast and School Lunch Validated with Observations: In-Person versus Telephone Interviews. J. Nutr. Educ. Behav..

[48] Scott A.R., Reed D.B., Kubena K.S., McIntosh W. (2007). Evaluation of a Group Administered 24-Hour Recall Method for Dietary Assessment. J. Ext..

[49] Beasley J., Riley W.T., Jean-Mary J. (2005). Accuracy of a PDA-based dietary assessment program. Nutrition.

[50] Byker Shanks C., Banna J., Serrano E.L. (2017). Food Waste in the National School Lunch Program 1978–2015: A Systematic Review. J. Acad. Nutr. Diet..

[51] Smith S.L., Cunningham-Sabo L. (2014). Food choice, plate waste and nutrient intake of elementary- and middle-school students participating in the US National School Lunch Program. Public Health Nutr..

[52] Paxton A., Baxter S.D., Fleming P., Ammerman A. (2011). Validation of the School Lunch Recall Questionnaire to Capture School Lunch Intake of Third- to Fifth-Grade Students. J. Am. Diet. Assoc..

[53] Miyawaki A., Lee J.S., Kobayashi Y. (2019). Impact of the school lunch program on overweight and obesity among junior high school students: A nationwide study in Japan. J. Public Health.

[54] (2020). Dietary Guidelines for Americans, 2020–2025.

[55] National Standards in the National School Lunch and School Breakfast Programs: Final Rule. Federal Register: 2012. https://www.govinfo.gov/content/pkg/FR-2012-01-26/pdf/2012-1010.pdf.

[56] Chen B., Kattelmann K., Comstock C., McCormack L., Wey H., Meendering J. (2021). Parenting Styles, Food Parenting Practices and Dietary Intakes of Preschoolers. Nutrients.

[57] Skinner J.D., Carruth B.R., Bounds W., Ziegler P.J. (2002). Children’s Food Preferences. J. Am. Diet. Assoc..

[58] Sharma S.V., Rashid T., Ranjit N., Byrd-Williams C., Chuang R.J., Roberts-Gray C., Briley M., Sweitzer S., Hoelscher D.M. (2015). Effectiveness of the Lunch is in the Bag program on communication between the parent, child and child-care provider around fruits, vegetables and whole grain foods: A group-randomized controlled trial. Prev. Med..

[59] Aydin G., Margerison C., Worsley A., Booth A. (2022). Parents’ Communication with Teachers about Food and Nutrition Issues of Primary School Students. Children.

[60] Kenney E.L., Cradock A.L., Long M.W., Barrett J.L., Giles C.M., Ward Z.J., Gortmaker S.L. (2019). Cost-Effectiveness of Water Promotion Strategies in Schools for Preventing Childhood Obesity and Increasing Water Intake. Obesity.

[61] Malik V.S., Pan A., Willett W.C., Hu F.B. (2013). Sugar-sweetened beverages and weight gain in children and adults: A systematic review and meta-analysis. Am. J. Clin. Nutr..

[62] Vinke P.C., Blijleven K.A., Luitjens M.H.H.S., Corpeleijn E. (2020). Young Children’s Sugar-Sweetened Beverage Consumption and 5-Year Change in BMI: Lessons Learned from the Timing of Consumption. Nutrients.

[63] Lowering Sodium in School Lunches. American Heart Association. https://www.heart.org/-/media/Files/About-Us/Policy-Research/Fact-Sheets/Access-to-Healthy-Food/INFOGRAPHIC-Lowering-Sodium-in-School-Foods.pdf.

[64] Connelly J., Palacios C., Campa A., Gonzalez N., De La Torre K., Panchana X. (2020). Sodium and Sugar Intake Comparison among Participants of the USDA National School Lunch Program Versus Non-Participants Based on 2015–2016 NHANES Survey. Curr. Dev. Nutr..

[65] Cohen J.F.W., Richardson S., Rimm E.B. (2019). Impact of the Updated USDA School Meal Standards, Chef-Enhanced Meals, and the Removal of Flavored Milk on School Meal Selection and Consumption. J. Acad. Nutr. Diet..

[66] Bergman E.A., Saade C., Shaw E., Englund T., Cashman L., Taylor K.W., Watkins T., Rushing K. (2014). Lunches Selected and Consumed from the National School Lunch Program in Schools Designated as Healthier US School Challenge Schools are More Nutritions than Lunches Brought from Home. J. Child Nutr. Manag..

[67] Panel on Macronutrients, Panel on the Definition of Dietary Fiber, Subcommittee on Upper Reference Levels of Nutrients, Subcommittee on Interpretation and Uses of Dietary Reference Intakes, Standing Committee on the Scientific Evaluation of Dietary Reference Intakes, Food and Nutrition Board (2005). Dietary Reference Intakes for Energy, Carbohydrate, Fiber, Fat, Fatty Acids, Cholesterol, Protein, and Amino Acids.

[68] Condon E.M., Crepinsek M.K., Fox M.K. (2009). School Meals: Types of Foods Offered to and Consumed by Children at Lunch and Breakfast. J. Am. Diet. Assoc..

[69] Williams C.L. (1995). Importance of Dietary Fiber in Childhood. J. Am. Diet. Assoc..

[70] Gan K., Tithecott C., Neilson L., Seabrook J.A., Dworatzek P. (2021). Picky Eating Is Associated with Lower Nutrient Intakes from Children’s Home-Packed School Lunches. Nutrients.

[71] Whitaker R.C., Wright J.A., Finch A.J., Deyo R.A., Psaty B.M. (1993). School lunch: A comparison of the fat and cholesterol content with dietary guidelines. J. Pediatr..

[72] (2015). Dietary Guidelines for Americans. 2015–2020.

